# Age and gender dependent heart rate circadian model development and performance verification on the proarrhythmic drug case study

**DOI:** 10.1186/1742-4682-10-7

**Published:** 2013-02-09

**Authors:** Kamil Fijorek, Nikunjkumar Patel, Łukasz Klima, Katarzyna Stolarz-Skrzypek, Kalina Kawecka-Jaszcz, Sebastian Polak

**Affiliations:** 1 Department of Statistics, Cracow University of Economics, Krakow, Poland; 2 Simcyp Limited, Sheffield, UK; 3 Department of Cardiology and Hypertension, Jagiellonian University Medical College, Krakow, Poland; 4 Unit of Pharmacoepidemiology and Pharmacoeconomics, Faculty of Pharmacy, Jagiellonian University Medical College, Krakow, Poland

**Keywords:** Heart rate, Circadian rhythm, Drugs cardiotoxicity, Regression modelling

## Abstract

**Background:**

There are two main reasons for drug withdrawals at the various levels of the development path – hepatic and cardiac toxicity. The latter one is mainly connected with the proarrhythmic potency and according to the present practice is supposed to be recognized at the pre-clinical (*in vitro* and animal *in vivo*) or clinical level (human *in vivo* studies). There are, although, some limitations to all the above mentioned methods which have led to novel *in vitro – in vivo* extrapolation methods being introduced. With the use of *in silico* implemented mathematical and statistical modelling it is possible to translate the *in vitro* findings into the human *in vivo* situation at the population level. Human physiology is influenced by many parameters and one of them which needs to be properly accounted for is a heart rate which follows the circadian rhythm. We described such phenomenon statistically which enabled the improved assessment of the drug proarrhythmic potency.

**Methods:**

A publicly available data set describing the circadian changes of the heart rate of 18 healthy subjects, 5 males (average age 36, range 26–45) and 13 females (average age 34, range 20–50) was used for the heart rate model development. External validation was done with the use of a clinical research database containing heart rate measurements derived from 67 healthy subjects, 34 males and 33 females (average age 33, range 17–72). The developed heart rate model was then incorporated into the ToxComp platform to simulate the impact of circadian variation in the heart rate on QTc interval. The usability of the combined models was assessed with moxifloxacin (MOXI) as a model drug.

**Results:**

The developed heart rate model fitted well, both to the training data set (RMSE = 128 ms and MAPE = 12.3%) and the validation data set (RMSE = 165 ms and MAPE = 17.1%). Simulations performed at the population level proved that the combination of the IVIVE platform and the population variability description allows for the precise prediction of the circadian variation of drugs proarrhythmic effect.

**Conclusions:**

It can be concluded that a flexible and practically useful model describing the heart rate circadian variation has been developed and its performance was verified.

## Introduction

The withdrawal of several marketed drugs in the last decade, extensive re-labeling and a high attrition rate, especially in the late stage of the drug development process, due to the QT prolonging liability or TdP occurrence, have all established that proarrhythmia assessment is a primary focus of regulatory bodies and the pharmaceutical industry. Thus careful evaluation of the proarrhythmic potential of an investigated compound is an integral element of the safety profile required for the approval of new drugs. There are several available models utilized for the assessment of drugs proarrhythmic potency at various levels of drug development. The most commonly utilized and probably most reasonable pathways at the non-clinical stage of development include *in vitro* models where the human ionic channels are heterologously expressed in non-human (i.e. XO, CHO) and human (i.e. HEK) cells, *in vivo*/*ex vivo* animal studies where suggested species are used for the drug influence evaluation at the cardiac level [1]. Novel techniques include the human stem cells derived cardiomyocytes which, however, still lack the standard methodology [2]. At the early clinical development stage the currently best available, yet costly and still not perfect thorough QT/QTc clinical studies (TQTs) [3] are introduced to assess the QT prolongation by the candidate drug as compared with placebo and positive control. Currently there is a translational gap in the quantitative extrapolation of the *in vitro* or animal studies’ outcomes to the human situation. There are some rules of thumb [4] and decision trees [5] available to qualitatively (yes/no) predict QT prolongation potential in humans from *in vitro* assays, particularly hERG channel inhibition assays. Moreover, there are no methods available to combine *in vitro* assay information from multiple ion channels (I_Na_, I_Ca_, I_Kr_, I_Ks_) with human physiological and demographic data to predict QT prolongation in humans. Physiologically based *in silico* methods have a potential to bridge the translation gap between the *in vitro* electrophysiological data gained from *in vitro*/*ex-vivo* studies and human QT liability. We have recently developed a so called “bottom-up” *in silico* platform (ToxComp) which combines a physiologically based electrophysiological model of human left ventricular cardiomyocytes and a database of human physiological, genotypic and demographic data enabling the prediction of the QT prolongation in humans based on the *in vitro* data [6,7]. ToxComp has been previously used to predict QT prolongation liability of various compounds [8,9]. Until now ToxComp has predicted the absolute QT prolongation liability of a compound assuming that the physiological parameter values are constant during the simulation, which limited its ability to simulate clinical scenarios. With the addition of the circadian variation model in ToxComp, the platform could potentially be employed in simulating clinical scenarios before conducting clinical studies with an aim to optimize the designs and reduce the chances of the failure of these costly and time intensive clinical studies.

A circadian rhythm is a biological process that displays an endogenous, entrainable oscillation of roughly 24 hours [10]. In humans there are multiple physiological processes which follow the circadian clock, including the heart rate (HR) [11]. In our study context, however, the circadian rhythm of the heart rate is one of the most important, since it may both influence the QT length and interfere with the drug effect [12]. The mathematical and statistical modelling of the circadian variation of the heart rate has been previously studied. Nakagawa and colleagues reported results of a study which involved 44 (but 20 were included into the analysis phase) healthy individuals. Subjects showed a significant circadian heart rate rhythm and based on the collected data the authors proposed a cosinor model to describe such phenomenon, although they did not differentiate between male and females which limits the practical use of the derived model [13]. Similar models, carrying identical limitations were proposed independently by Massin and colleagues and Li and colleagues [14,15]. 57 healthy children and 115 healthy, non-smoking adults were included respectively and the 24-hours ECG measurements were used to derive the regression models. Probably the most detailed analysis of the heart rate variation was presented by Bonnemeier et al. [16]. A large group of 166 healthy individuals (81 females, 85 males) characterized by a wide age range (20–70 years) was studied. The authors analysed differences of heart rate circadian profiles for hourly aggregated measurements in a groups stratified by age decades, separately for female and male subjects. The authors did not, however, model their data. There is also a large number of publications where circadian heart rate variation is discussed among various subpopulations (athletes, truck drivers, welders) and diseases (myotonic dystrophy, angor patients) [17-20]. Although to our best knowledge none of them proposed a model flexible enough and described in enough detail to be directly applicable for the generation of a virtual human population where heart rate is an attribute specific for every individual, and this was the main reason we decided to develop a new model.

### Aims of the study

We aimed to develop and validate a model describing the gender and age dependent circadian rhythm of the heart rate in European Caucasian healthy subjects. Additional aims included quality and usability assessment of the model when incorporated into the ToxComp platform. Its usability, in combination with other parameters describing demographic (age, gender), physiological (i.e. cardiomyocytes characteristics, plasma ions concentration) and genetic variability in a population, was tested in the virtual clinical trial.

## Materials and methods

### PhysioNet data set description

The analyzed data set was obtained from the PhysioBank, which is an archive of digitized sets of data reflecting physiological signals. The data warehouse contains over 50 various freely available databases, and for modelling purposes the MIT-BIH Normal Sinus Rhythm Database was used [21]. There were a total of 18 subjects, 5 males (average age 36, range 26–45) and 13 females (average age 34, range 20–50). For each subject up to 24 hours of RR recordings were available. Subjects, on average, had 94,440 individual RR measurements (range 73,300-115,900). In order to decrease the significant computational burden it was necessary to reduce the number of RR measurements per subject. RR averaging in one-hour, half-hour and 15-minute ‘time windows’ was found inadequate due to its variability reduction property. Stable results were obtained after sampling RR measurements every 1 minute.

### Validation data set

Model validation was performed with the use of a completely independent data set. Data was derived from Cracow’s clinical research database (1^st^ Department of Cardiology and Hypertension, Jagiellonian University Medical College). There were a total of 67 healthy subjects in the validation data set, 34 males and 33 females (average age 33, range 17–72). Validated oscillometric SpaceLabs 90207 monitors (Redmond, WA, USA) fitted with the appropriate cuff size were programmed to obtain readings every 15 minutes from 08.00 to 22.00 and every 30 minutes from 22.00 to 08.00. Each reading included systolic/diastolic blood pressure, mean arterial pressure and heart rate. Subjects, on average, had 71 heart rate measurements (range 35–99). RR interval length was calculated based on the heart rate measurement results.

### Model usability testing

To test the usability of the implemented circadian rhythm model within the ToxComp platform, we have chosen moxifloxacin (MOXI), the most commonly used positive control in TQT studies as the model compound. The ToxComp system was used to simulate the drugs triggered ECG modification. The ToxComp platform combines a physiologically based electrophysiological model of human left ventricular cardiomyocytes (ten Tusscher - TNNP) and a database of human physiological, genotypic and demographic data enabling the prediction of the QT prolongation in humans based on the *in vitro* data [7,22]. To account for the heterogeneities in ionic currents between endocardial, midmyocardial and epicardial cells 1D fibre paced at the epicardial side was constructed. The 50:30:20 distribution of the endo-, mid- and epicardial cells was used together with a diffusion coefficient equal to 0.0016 cm^2^/ms. The Forward Euler method was used to integrate model equations. Integration results were used to calculate a pseudo-ECG. First and last QRS were excluded from the pseudo-ECG. A space step and a time step were set to Δx=0.01 mm and Δt=0.01 ms, total simulation time was set to 10,000 ms.

To account for the drugs triggered ionic currents modifications a specific equation describing the current of interest was multiplied by the inhibition factor accordingly to the *in vitro* values provided by the literature search, which described the concentration dependent ionic current inhibition. The inhibition factor was calculated with the use of the Hill equation [Equation 1].

(1) $$\text{Inhibition}\;\text{Factor}=\frac{1}{1+{\left(IC_{50}/\text{DRUG}\;\text{CONCENTRATION}\right)}^{n}}$$

where:

IC_50_ - concentration responsible for the 50% inhibition of the ionic current

n - Hill equation parameter

DRUG CONCENTRATION - active drug concentration [μM]

The population variability of other parameters was mimicked by applying the virtual population generator as described previously [23,24]. The circadian heart rate variability was introduced into a simulation by the use of the model described below.

3.8 μM of MOXI was used for the operational concentration, mimicking the average maximum free plasma concentration (C_max_) after a 400 mg oral dose by correcting the total C_max_ concentration obtained from the available literature with human plasma protein binding [25,26]. The IC_50_ values for various cardiac ion channels were obtained from the tox-database.net system and are presented in Table 1[27]. For the simulation studies only I_Kr_ data was used (with Hill equation parameter n = 1) since for the tested MOXI concentration I_Na_ and I_Ca_ inhibition were negligible.

**Table 1:** **Moxifloxacin IC**_**50**_**values for various cardiac ionic currents**

**Current**	**IC**_**50**_**[μM]**	**Reference**
I_Kr_	29	[28]
I_Na_	127.2	[29]
I_Ca_	168.9	[29]

The ToxComp simulations, at a MOXI concentration equal to C_max_ at different times of the day (4.00, 8.00, 12.00, 16.00, 20.00, and 24.00), were performed in triplicate trials of 20 individuals (total 3×20=60) with an equal number of male and female subjects (10/10 in each study). Baseline QT and QTcF (QT interval corrected by heart rate using Fridericia correction method [30]) were also obtained for each subject at the above defined time points of the day by simulating a response at MOXI concentration equal to zero. This allowed us to apply two baseline correction formulas i.e., (1) Single point baseline correction when the average baseline QTcF estimates from all subjects was used to obtain baseline corrected QTcF (ΔQTcF) and (2) Individualised baseline correction (ΔQTcF_i_) when baseline QTcF at a given time of day for each individual subject was used to calculate ΔQTcF. The developed circadian effect model was validated by comparing the simulated results with respective clinical outcomes obtained using single point and individualised baseline correction formula.

## Results

### Data modelling

The aim of this section is to describe the steps undertaken to create a multivariate linear regression model of the relationship between RR (dependent variable) and a set of independent variables which were comprised of Age, Sex and Time of the day (abbreviated as Hour). In addition to the model development, the simulation of RR values from the model was given attention. During the model development phase it was found that the distribution of RR data was mildly skewed to the right. This issue was dealt with the use of log-transformation of RR measurements.

### RR model

The preliminary model included dummy variables for each Hour, dummy variable for Sex, quadratic effect of Age and all pairwise interactions. Many terms in this preliminary model were found not significant, as indicated by the robust t-tests for model coefficients. The robust *t*-test takes into account the dependence between observations coming from the same subject. Non-significant terms (p-value>0.05) were dropped from the model sequentially one by one. Additionally it was found that the Hour variable may be modelled more parsimoniously by a linear combination of sine and cosine functions. The final model received the following form:

(2) $$\begin{array}{l} \text{log}\left(\text{RR}\right)=\beta_{0}+\beta_{1}Sex+\beta_{2}Age+\beta_{3}Age^{2}+\beta_{4}\sin\left(\frac{2\pi }{24}\text{Hour}\right)\\ \;+\beta_{5}\text{cos}\left(\frac{2\pi }{24}\text{Hour}\right)+\beta_{6}\sin\left(\frac{2\pi }{24}\text{Hour}\right)\times \text{Sex}+\beta_{7}\text{cos}\left(\frac{2\pi }{24}\mathrm{Hour}\right)\times \text{Sex}+\varepsilon ,\;\varepsilon ∼N\left(0,\sigma \right), \end{array}$$

where:

β – regression coefficient,

ε – normally distributed error term,

*σ* – standard deviation of error term,

Sex – 1 for males, 0 for females,

Age – age in years,

Hour – value from 0–24 range.

The model parameters were estimated using the maximum likelihood method in the R system for statistical computing and are given below [31]:

(3) $$\begin{array}{l} \hat{\text{log}\left(\text{RR}\right)}=7.163+0.0961\;\text{Sex}-0.0243\;\text{Age}+0.00027\;Age^{2}\\ \;+0.1055\;\sin\;\left(\frac{2\pi }{24}\text{Hour}\right)+0.0664\;\text{cos}\;\left(\frac{2\pi }{24}\text{Hour}\right)\\ \;-0.0155\;\text{sin}\;\left(\frac{2\pi }{24}\text{Hour}\right)\times \text{Sex}+0.0608\;\text{cos}\;\left(\frac{2\pi }{24}\text{Hour}\right)\times \text{Sex},\sigma \\ \;=0.15 \end{array}$$

**Figure 1:**
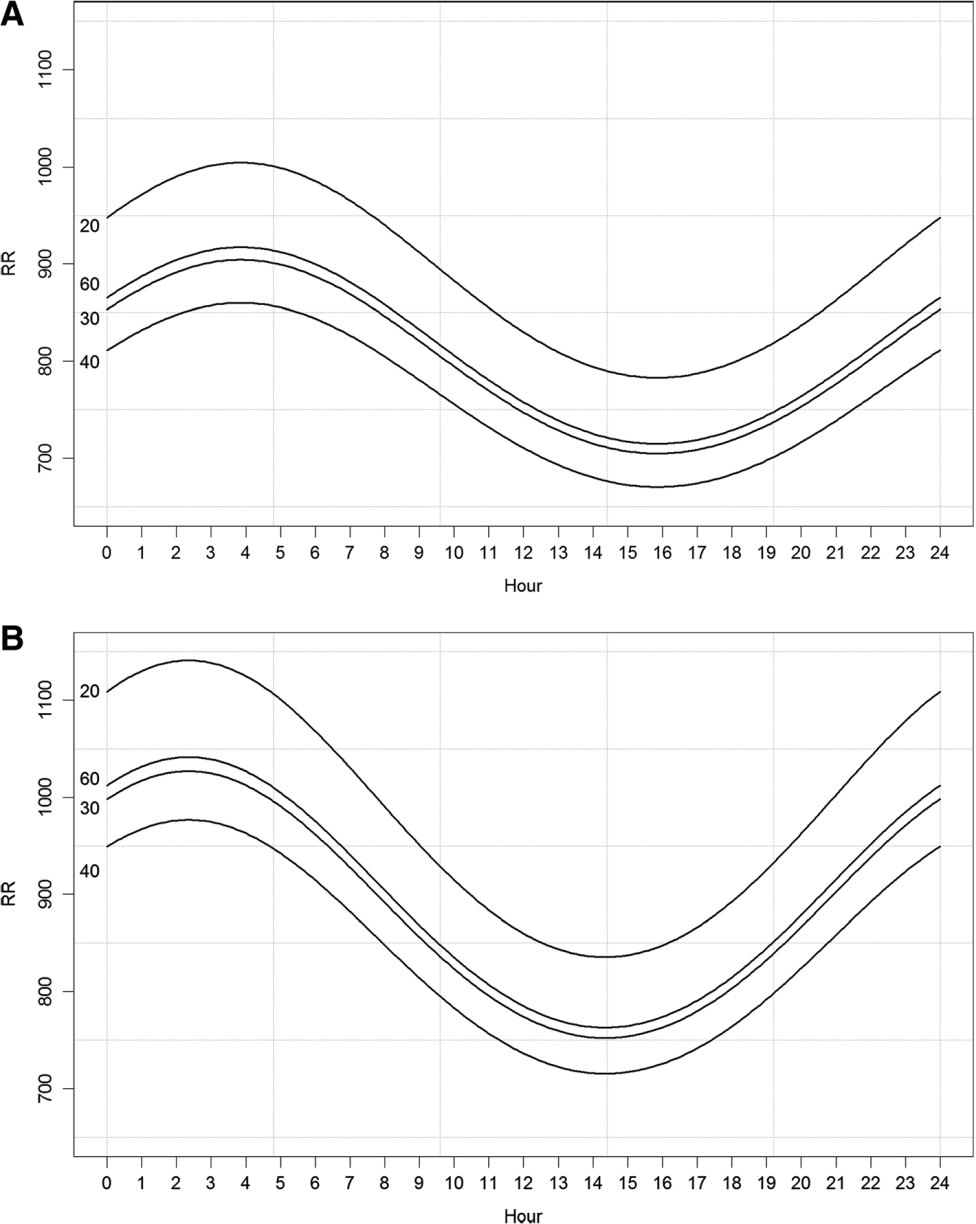
Graphical representation of the RR model for females (A) and males (B) respectively, left inner axis is age.

All coefficients were statistically significant. Figure 1 shows the graphical representation of the model.

The visual predictive checks showed an overall good fit of the model to the data. The dispersion of residuals did not exhibit dependence upon any of the explanatory variables. The coefficient of determination R^2^ = 0.39, i.e. 39% of the variation observed in log(RR) can be explained by the estimated regression equation.

**Figure 2:**
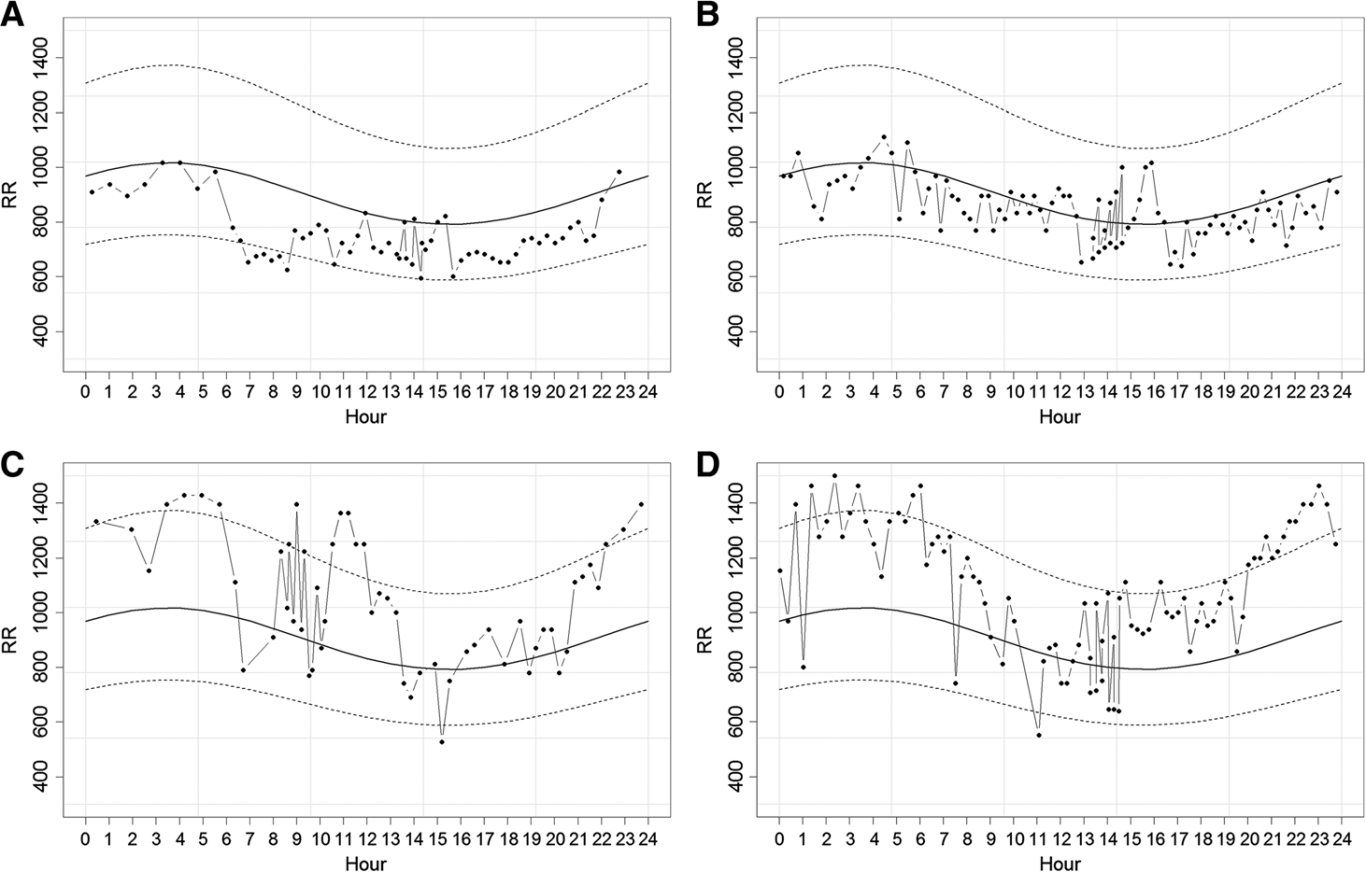
Representative two best (A - Female 41 years old, B - Male 36 years old) and two worst (C - Female 27 years old, D - Male 30 years old) fitted cases from the validation data set (dashed lines – 95% prediction bands).

For the PhysioNet data set RMSE (Root Mean Squared Error) = 128 ms and MAPE (Mean Absolute Percentage Error) = 12.3%. In the case of the validation data set RMSE = 165 ms and MAPE = 17.1%. Figure 2 shows the representative two best and two worst fitted cases from the validation data set.

It is a well-known fact that estimators derived by the method of maximum likelihood have many desirable properties, on the other hand ML estimators are sensitive to the presence of outliers in the data. In order to check the stability of estimates the regression parameters were additionally estimated using a wide range of, so called, robust regression estimators. Parameter estimates were virtually unchanged from the maximum likelihood ones.

The residuals (

$$\hat{\varepsilon }$$)

within each subject showed strong temporal dependence. Consequently the stationary autoregressive model of the following form was fitted:

(4) $${\hat{\varepsilon }}_{t}=\overset{P}{\underset{p=1}{\sum }}\alpha_{p}{\hat{\varepsilon }}_{t-p}+\eta ,\eta ∼N\left(0,\tau \right)$$

where:

α – autoregression coefficient

*η* – normally distributed error term

*τ* – standard deviation of error term

P – order of autoregression

It was found that only a large value of P (P = 180) was able to account for the long memory observed in the data and gives a satisfactory fit. The estimate of the standard deviation of error term was 0.096.

The following algorithm facilitates the generation of random RR values from the estimated model:

1. Generate random sequence from equation [Eq. 4].

2. Choose the values of explanatory variables and using [Eq. 3] compute

$$\hat{\text{log}\left(\text{RR}\right)}$$

at the selected time points.

3. Add the computed

$$\hat{\text{log}\left(\text{RR}\right)}$$

to values generated in Step 1 at the selected time points, exponentiate the result.

An electronic supplement (Additional file 1) to the article contains a fully functional Excel implementation of the model.

### Model usability results

**Figure 3:**
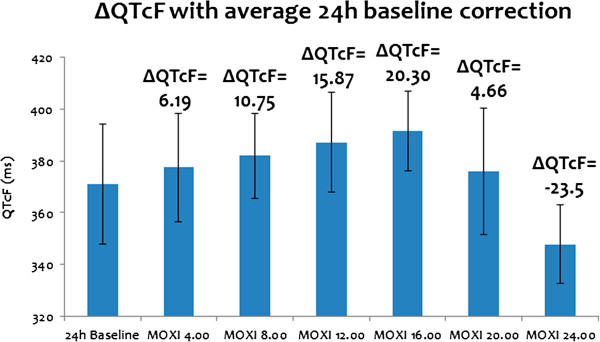
ΔQTcF at MOXI concentration of 3.8 mM at different times of day presented as an average value ±SD.

**Figure 4:**
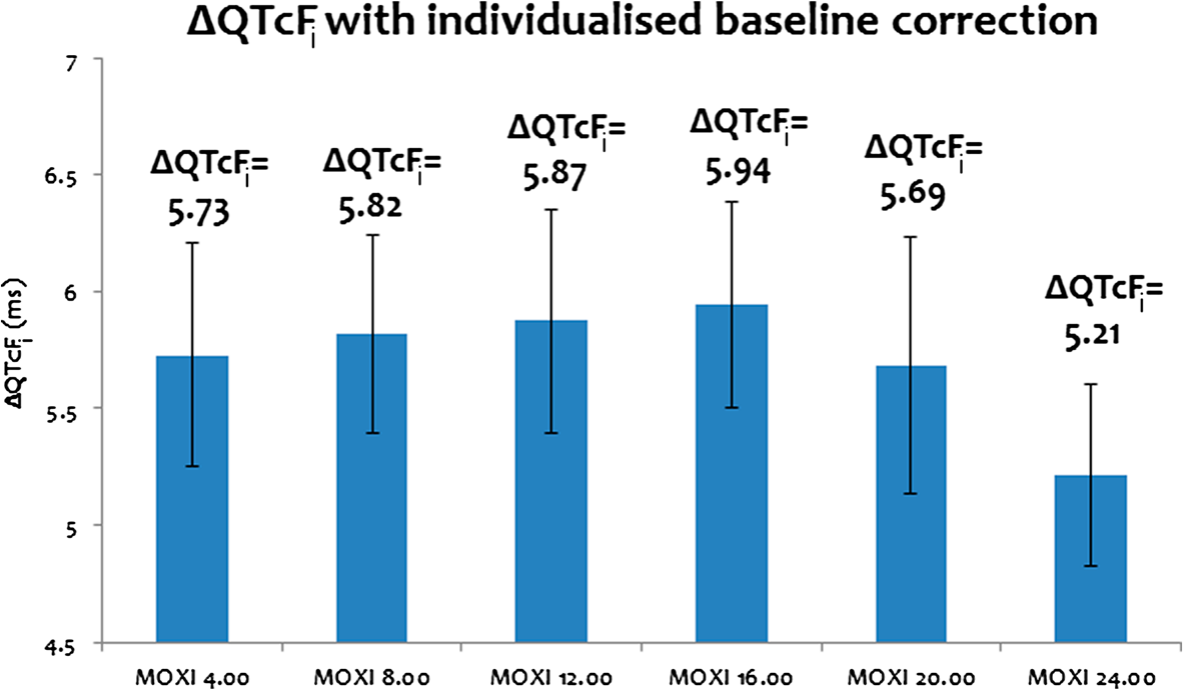
**ΔQTcF**_**i **_**with individualized and circadian baseline correction presented as an average value ±SD.**

Figures 3 and 4 present the results of the model usability testing. Two sets of data obtained after running the virtual study as described in the ‘Materials and methods’ section were obtained.

QT response at a MOXI concentration of 3.8 μM at different times of the day was simulated using the ToxComp platform. When single point baseline correction was applied, the ΔQTcF obtained at different times of the day at a MOXI concentration of 3.8 μM is shown in Figure 3. Generally, during TQT studies an oral dose of MOXI 400 mg is given in the morning, hence C_max_ is obtained between morning and noon i.e. between 8.00 to 12.00. At 8.00 and 12.00, the simulated ΔQTcF were respectively 10.75 ms and 15.87 ms, which is consistent with the clinically observed ΔQTcF of 10–15 ms [32]. When an individualized baseline with circadian correction was applied, ΔQTcF_i_ of 5–6 ms was obtained at all times of day which is also consistent with the clinically observed ΔQTcF_i_ of 6–7 ms [21].

## Discussion

The obtained results indicate that the implemented heart rate circadian rhythm model within the ToxComp platform is able to simulate the circadian variability of a model drug. Comparison with the published clinical trial results for the model drug suggest that the ToxComp system, with the built-in heart rate model, is able to realistically represent cardiac electrophysiological drug effect with its variability, regardless of the correction method. However, as there are a large number of parameters describing the physiological variability in the virtual population generator ToxComp module, it is hard to precisely assess the net influence of the heart rate on the system output, and a separate study will be run for such a purpose. Although results of the simulations run without the use of a heart rate model, i.e. with the use of a constant value of HR during the day, suggested that an important component is missing (internal not-published results).

The goodness-of-fit metrics indicated a good fit to the data, e.g. Mean Absolute Percentage Error equals to 12.3%. It could, although, suggest that the model overfits the data. This was however contradicted by the validation results with MAPE equals to 17.1%. The result was obtained despite the differences in the age and sex distribution of the subjects between the training and validation data sets.

The main limitation of the model is the size of the training data set. Despite the fact that every subject gave rise to thousands of measurements, the number of independent subjects is still only 18. The future research will aim to combine data from experiments which acquire sparse RR data, e.g. every 20–40 minutes, with dense PhysioBank data.

## Conclusions

It can be concluded that a flexible and practically useful model describing the heart rate circadian variation has been developed. Its structure allows for easy implementation, which is greatly facilitated by the provided electronic supplement, and its use in simulation studies.

## Abbreviations

CHO: Chinese Hamster Ovary cells; HEK: Human Embryonic Kidney cells; IVIVE: *In vitro* – *in vivo* extrapolation; MAPE: Mean Absolute Percentage Error; ML: Maximum likelihood; MOXI: Moxifloxacin; QTcF: QT interval corrected for heart rate with the Fridericia correction;ΔQTcF: Difference between the average of baseline QTcF of all subjects and QTcF in the presence of a drug;ΔQTcFi: Difference between the baseline QTcF of each subject at a given time of day and QTcF in the presence of a drug for the same subject; R2: Coefficient of determination;RMSE: Root Mean Squared Error; RR: Interval from the onset of one QRS complex to the onset of the next QRS complex; SD: Standard deviation; TdP: Torsade de Pointes; TQT: Thorough QT study; XO: Xenopus Oocytes.

## Competing interests

The authors declare that they have no competing interests.

## Authors’ contributions

KF and NP participated equally in the study conductance, KF – model development, NP – model validation. Study idea and methodology development come from SP and KF. KF, NP and SP were equally involved in the manuscript writing. ŁK, KS-S, KK-J were responsible for the clinical data gathering and control from the clinical point of view. All authors read and approved the final manuscript.

## Supplementary Material

Additional file 1 Electronic Supplement 1. Click here for file
